# In Vivo Hypoglycemic Effects, Potential Mechanisms and LC-MS/MS Analysis of *Dendropanax Trifidus* Sap Extract

**DOI:** 10.3390/nu13124332

**Published:** 2021-11-30

**Authors:** Ahreum Lee, Yuki Sugiura, Ik-Hyun Cho, Noriko Setou, Eugene Koh, Gyun Jee Song, Seungheun Lee, Hyun-Jeong Yang

**Affiliations:** 1 Korea Institute of Brain Science, Seoul 06022, Korea; dkfma5025@hanmail.net (A.L.); brainedu2020@gmail.com (S.L.); 2 Department of Biochemistry and Integrative Medical Biology, School of Medicine, Keio University, Tokyo 160-8582, Japan; yuki.sgi@gmail.com; 3 Department of Convergence Korean Medical Science, College of Korean Medicine, Kyung Hee University, Seoul 02447, Korea; ihcho@khu.ac.kr; 4 Department of Disaster Psychiatry, Fukushima Medical University, Fukushima 960-1295, Japan; setou@fmu.ac.jp; 5 Temasek Life Sciences Laboratories, Singapore 117604, Singapore; eugene@tll.org.sg; 6 Department of Medical Science, Catholic Kwandong University College of Medicine, Gangneung 25601, Korea; gyunjeesong@gmail.com; 7 Department of Integrative Health Care, University of Brain Education, Cheonan 31228, Korea

**Keywords:** *Dendropanax trifidus*, *Dendropanax morbiferus*, in vivo toxicity, blood glucose, LC-MS/MS, AMPK

## Abstract

Extracts of medicinal plants have been widely used to benefit human health. *Dendropanax morbiferus* (DM) has been well-studied for its anti-inflammatory and anti-oxidative effects, while *Dendropanax trifidus* (DT) is a lesser-known ecotype phylogenetically similar to DM, which has received significantly less attention. Studies thus far have primarily focused on leaf and bark extracts of DM, and not much is yet known about the properties of either DM or DT sap. Therefore, here we performed in vivo toxicity and efficacy studies, in order to assess the biological effects of DT sap. To establish a safe dosage range, single dose or two-week daily administrations of various concentrations were performed for ICR mice. Measurements of survival ratio, body/organ weight, blood chemistry, histochemistry and Western blots were performed. A concentration of ≤0.5 mg/g DT sap was found to be safe for long-term administration. Interestingly, DT sap significantly reduced blood glucose in female mice. In addition, increasing concentrations of DT sap decreased phosphorylated (p) insulin receptor substrate (IRS)-1(ser1101)/IRS-1 in liver tissues, while increasing pAMP-activated protein kinase (AMPK)/AMPK in both the liver and spleen. To analyze its components, liquid chromatography-tandem mass spectrometry of DT sap was performed in comparison with *Acer saccharum* (AS) sap. Components such as estradiol, trenbolone, farnesol, dienogest, 2-hydroxyestradiol and linoleic acid were found to be highly enriched in DT sap compared to AS sap. Our results indicate DT sap exhibits hypoglycemic effects, which may be due to the abundance of the bioactive components.

## 1. Introduction

*Dendropanax* is a genus of flowering plants in the family *Araliaceae* [1]. Although both *Dendropanax morbiferus* (DM) and *Dendropanax trifidus* (DT), which belong to this family, are native to eastern Asia, each specific habitat is distinct; the southern coast of the Korean Peninsula for DM [2,3,4], and Japan for DT [2,5]. However, according to previous PCR-Random Amplified Polymorphic DNA (RAPD) analysis of DM and DT, their genetic differences were not clear [2]. In a study of the fruit, sap color and leaf external morphologies of DM and DT, a clear distinction between the two was also not possible [6]. In another study which investigated 13 taxa of *Dendropanax* of Korea, Japan and China, the morphological features of DM and DT were insufficiently distinguishable, therefore the authors suggested to classify them as conspecific taxa under DT [7]. In a phylogenetic analysis using nuclear ribosomal DNA internal transcribed spacer (ITS) region sequences, DM was found to share 99.4% ITS region similarity with DT [8]. The accumulation of these studies warrants that these two groups be treated as the same taxonomic species (National Institute of Biological Resources), but more detailed molecular phylogenetic studies may be required.

There is little research on the efficacy of DT. It has only been reported as causing allergic contact dermatitis in Japan [5,9]. However, it has been historically used as a medicinal herb to treat various conditions such as migraine, dysmenorrhea and dermatopathy in Korean traditional medicine [10], and various effects such as antioxidant, anti-diabetic, anti-inflammatory and anti-complement activities have been reported for leaves, branches, barks and stems of DM [11,12,13,14]. Combined extracts of DM leaves and branches exhibited antioxidant activity, viability and α-glucosidase inhibitory effects on 3T3-L1 cells [11]. DM bark extracts contained monoterpene sugar derivatives of long-chain compounds (cis-6-oxogeran-4-enyl-10-oxy-O-β-arabinopyranosyl-4′-O-β-arabinopyranosyl-2″-octadec-9‴,12‴,15‴-trienoate and geran-3(10)-enyl-1-oxy-O-β-arabinopyranosyl-4′-O-β-arabinopyranosyl-2″-octadec-9‴,12‴,15‴-trienoate), which exhibited antioxidant activities [12]. In streptozotocin (STZ)-treated type 1 diabetic rats, DM stem extracts reduced blood glucose (GLU) levels, escape latency in the Morris water maze test, microglial activation and inflammation [13]. DM leaf extract components exhibited in vitro anticomplement activities [14], which play an important role in host defense. In comparison with other parts of DM (or DT), the properties and effects of its sap are not as well-studied. Only anti-cancer effects of its exosomes have been recently described [15,16]. However, toxicity studies or other functional effects of DT sap itself have not yet been reported. 

The blood GLU lowering effects of DM extracts suggests that it may modulate GLU metabolism, but the biological mechanisms behind this are not well characterized. Blood GLU levels are regulated primarily by the interplay of the hormones insulin and glucagon. Secretion of insulin by pancreatic β-cells stimulates the uptake of glucose from the bloodstream, reducing blood GLU levels, and vice versa for glucagon. In the canonical insulin-signaling pathway, insulin binds and activates insulin receptor substrate (IRS), triggering Akt activation in its downstream signaling pathway [17]. Akt activation stimulates the translocation of GLU transporter (GLUT) membrane transporters to the plasma membrane, which promotes the influx of glucose into the cell [18]. AMP-activated protein kinase (AMPK) is a central regulator of cellular homeostasis and mediates a variety of metabolic pathways [19]. In particular, AMPK activation stimulates the expression and translocation of GLUT1 and GLUT4 to the plasma membrane [20]. In addition, AMPK-mediated glucose metabolism includes a wide range of pathways including also gluconeogenesis, glycolysis and glycogen storage [19].

Here, we investigated the in vivo toxicity thresholds of DT sap, and performed measurements of blood chemistry in DT sap-treated ICR mice. In order to better understand the hypoglycemic effects of DT sap observed here, we also performed Western blots on various signaling molecules involved in insulin and AMPK-mediated signaling. Furthermore, we used liquid chromatography-tandem mass spectrometry (LC-MS/MS) to elucidate the chemical composition of DT sap, providing a rich source of information for future studies. 

## 2. Materials and Methods

### 2.1. Mice

Eight-week-old mice of CrljOri:CD1 (ICR) lines were purchased from ORIENT BIO Inc. (Seongnam, Korea). After 1 week of rest, 9-week-old mice were used for the experiments. Mice were kept on a 12-h light–dark cycle with light period of 8:00–20:00 in a standard specific pathogen-free environment. Mice were provided with regular chow and water ad libitum. Each animal was anaesthetized by carbon dioxide inhalation before sampling. All experiments were performed in compliance with the relevant laws and institutional guidelines and were approved by the University of Brain Education’s Animal Care and Use Committee (Approval number: 2018-AE-01Y). Mouse number used in each experiment is indicated in Table S1.

### 2.2. Dendropanax trifidus Sap Preparation

*Dendropanax trifidus (Thunb.) Makino ex H.Hara* was collected from the forest in Simasi, Mieken, Japan and registered at Ibaraki Nature Museum: voucher number INM-2-212778. DT sap was dissolved in 100% EtOH, freeze dried and stored at −20 °C. The freeze-dried final product with viscosity was dissolved in EtOH to make a stock solution of 1.4 or 0.5 g/mL and stored at −20 °C. The extract is referred as DT sap in this paper. 

### 2.3. In Vivo Toxicity Test

**Figure 1:**
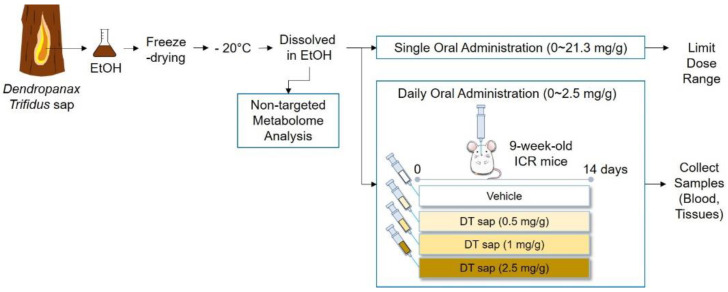
Experimental design. Experimental procedures are briefly described in the scheme. *Dendropanax Trifidus* sap was extracted in 100% EtOH, freeze-dried and maintained at −20 °C. Its EtOH-dissolved solution was subjected to in vivo mouse studies. A single oral administration was conducted to limit the dose range of DT sap (Figure S1). Based on this information, daily oral gavage of three concentrations was performed for 14 days. Survival rate and body weights were measured at day 0, 1, 3, 7 and 14 (Figure 2). Organ weight measurement (Figure 3), blood chemistry (Figure 4), histochemistry (Figure 5) and Western blot analysis (Figure 6) were performed on the samples of day 14. In order to analyze the chemical components of DT sap, it was subjected to non-targeted metabolome analysis (Figure 7, Table 1).

To determine the optimal concentration of DT sap for in vivo administration, short-term effects of DT sap on survival as well as body and organ weights were examined when administered in a single dose of various concentrations (0, 1.2, 2.5, 4.8, 10.3, 21.3 mg/g, DT sap weight/body weight) to 9-week-old ICR mice by oral gavage. After the single injection, the mice were observed for several hours, and body weight was measured once per day for an initial 3 days. At day 20, body weight was measured again, and mice were sacrificed, thereafter organ (liver, spleen, kidney, lung, heart = and adrenal gland) weights were measured separately (Figure S1). We further examined the effects of multiple injections with several lower concentrations (0, 0.5, 1 and 2.5 mg/g, DT sap weight/body weight) every day for 14 days to 9-week-old ICR mice by oral gavage. Mouse body weight was measured at day 0, 1, 3, 7 and 14, and the mice were sacrificed at day 14 to obtain organs for weight measurements, blood chemistry, Western blots and histochemistry. Before the sacrifice, all animals were fasted overnight (Figure 1). 

### 2.4. Blood Chemistry

DT sap extracts of the indicated concentrations were given to ICR mice for 14 days by daily oral gavage. Whole blood was withdrawn from the heart and maintained at room temperature (RT) for 30 min to clot, then centrifuged at 2000× *g* for 10 min in a refrigerated centrifuge, and the resulting supernatant (serum) was subjected to blood chemistry analysis. In the serum, the following parameters were measured by using a BS-200 Chemistry Analyzer (Mindray, China): GLU, glutamic pyruvate transaminase (GPT), glutamic oxalacetic transaminase (GOT), total protein (TP), blood urea nitrogen (BUN), alkaline phosphatase (ALP), lactate dehydrogenase (LDH), triglyceride (TG), cholesterol (CHOL), low-density lipoprotein (LDL) and high-density lipoprotein (HDL).

### 2.5. Western Blot

Western blot was performed as previously described [21]. Briefly, tissues were lysed in RIPA lysis buffer (WSE-7420, ATTO, DAWINBIO Inc, Hanam, Korea), and centrifuged at 15,000 RPM for 15 min. The supernatant was quantified by Bradford assay, diluted with 5 × Sample buffer, boiled and subjected to sodium dodecyl sulfate polyacrylamide gel electrophoresis (SDS-PAGE) for protein separation. Proteins were transferred onto PVDF membranes and blocked with EZBlock Chemi (AE-1475, ATTO, Tokyo, Japan) for 1h at RT. Membranes were incubated with the primary antibody for overnight at 4 °C, washed and incubated with secondary antibody for 1h at RT, and washed. Proteins were visualized by Super Signal West Pico PLUS Chemiluminescent Substrate (34580, ThermoFisher Scientific, Waltham, MA, USA) and captured by Amersham Imager 600 (GE Healthcare, Chicago, IL, USA). Images were analyzed by using Image J. For blotting, rabbit antibodies to the following antigens were purchased from Cell Signaling Technology (Danvers, MA, USA): phospho-IRS-1 (Ser1101) (2385), IRS-1 (2382), phospho-AMPKa (2531), AMPKa (2532), phosphor-Akt (4060) and Akt (9272). Rabbit antibodies to beta actin, Glut1 (ab652) and Glut4 (ab654) were purchased from Abcam (Cambridge, UK). For secondary antibody, horseradish peroxidase conjugate goat anti-rabbit IgG (Jackson Immunoresearch, West Grove, PA, USA) was used.

### 2.6. Histopathology

To assess the histopathological changes of the kidney and liver after DT sap extract administration, the ICR mice were anesthetized with CO_2_ and then perfused intracardially with saline and cold 4% paraformaldehyde in 0.1 M phosphate buffer (PB, pH 7.4). The kidney and liver were removed, post-fixed, washed, dehydrated and embedded with melted paraffin wax [22]. Paraffin blocks were cut into 5-μm-thick sections using a Leica RM 2155 microtome (Leica Biosystems, Wetzlar, Germany). The paraffin sections were stained with hematoxylin–eosin (H&E) dye as previously described [22]. Images of stained sections were visualized and captured using a DP70 digital light microscope system (Olympus, Tokyo, Japan). The kidney and liver morphology were observed under 40x magnification.

### 2.7. Non-Targeted Metabolome Analysis

Ethanol extracts of DT sap and *Acer saccharum* (AS) sap (Alleghanys Maple Farms Inc., Saint-Pacome, QC, Canada) were analyzed as previously described [23]. Briefly, for non-targeted analysis, metabolome data obtained by orbitrap-type MS (Q-Exactive Focus, Thermo Fisher Scientific, San Jose, CA, USA) connected to a HPLC (Ultimate3000 system, Thermo Fisher Scientific) with the discovery HS F5-3 column or an IC (ICS-5000+, Thermo Fisher Scientific) with the IonPac AS11-HC, 4-μm particle size column were analyzed. A Compound Discoverer 2.0 (Thermo Fisher Scientific) was used for the non-targeted metabolomics workflow. In brief, this software first aligned the total ion chromatograms of different samples along the retention time. Then, the detected features with an intensity of no less than 100,000 and an S/N larger than five in each set of data were extracted and merged into components. The resulting compounds were identified by both (i) formula prediction based on accurate m/z value and isotope peak patterns and (ii) MS/MS structural validation. Moreover, formula predicted signals were assigned into candidate compounds by database search (Chemspider database; http://www.chemspider.com/, accessed on 19 December 2019). 

### 2.8. Statistics

Statistical analyses were performed using one way analysis of variance (ANOVA) with post hoc tests (Holm–Sidak method or Dunn’s method), Kruskal–Wallis one way ANOVA on Ranks and Student’s *t*-test. 

## 3. Results

### 3.1. Effects on Survival Ratio and Body/Tissue Weight by a Single Administration of Dendropanax trifidus Sap

For assessing the in vivo toxicity of DT sap, a single administration regime was performed first in 9-week-old ICR mice by oral gavage, in order to limit the dose range. For the single administration (Figure S1), DT sap of a wide range of concentrations (0, 1.2, 2.5, 4.8, 10.3 and 21.3 mg/g for DT sap weight/mouse body weight) were separately injected. Mice injected with the highest concentration of 21.3 mg/g died at day 2; however, mice administered with concentrations lower than 21.3 mg/g survived till the end of the experimental period (i.e., day 20 after the administration) (Figure S1A). There were no significant changes in body weight for the initial three days after the injection (Figure S1B). However, in the measurement at day 20 post-administration, the concentration of 10.3 mg/g increased total body weight and spleen weight compared to other concentrations (Figure S1C,E). In the concentrations of ≤4.8 mg/g, total body weight and organ weights were not changed by the injection (Figure S1B–I).

### 3.2. Effects on Survival Ratio and Body/Tissue Weight by a Multiple Administration of Dendropanax trifidus Sap

**Figure 2:**
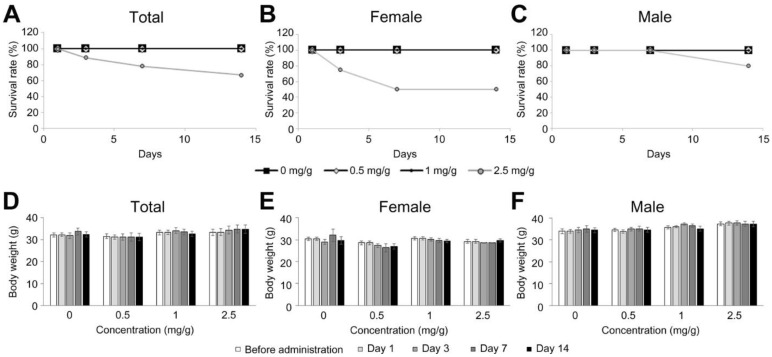
Effects on survival rate and body weight by *Dendropanax trifidus* (DT) sap daily administration in mice for two weeks. DT sap of the indicated concentrations was given to 9-week-old ICR mice by oral gavage every day for 14 days. (**A**–**C**) Survival rate at day 1, 3, 7 and 14 since the initial administration with respect to concentration of 0, 0.5, 1 and 2.5 mg/g (DT sap weight/body weight). (**D**–**F**) Body weight at day 0 (before administration), 1, 3, 7 and 14 with respect to concentration. (**A**,**D**) Total; (**B**,**E**) female; (**C**,**F**) male. N = 4–5 mice per group. Bars indicate mean ± s.e.m.

Therefore, for the multiple administration regime, the highest concentration used among the tested concentrations was set lower than 4.8 mg/g (i.e., 2.5 mg/g). The indicated concentrations (0, 0.5, 1 and 2.5 mg/g) were applied every day by oral gavage for 14 days (Figure 2, N = 4 females and 5 males/concentration). At day 14 (i.e., end of the experimental period), the survival ratio was 100% for all the concentrations except the highest concentration (50% for female and 75% male at 2.5 mg/g, Figure 2A–C). There were no significant changes in mouse body weight by concentration and time (Figure 2D–F). 

**Figure 3:**
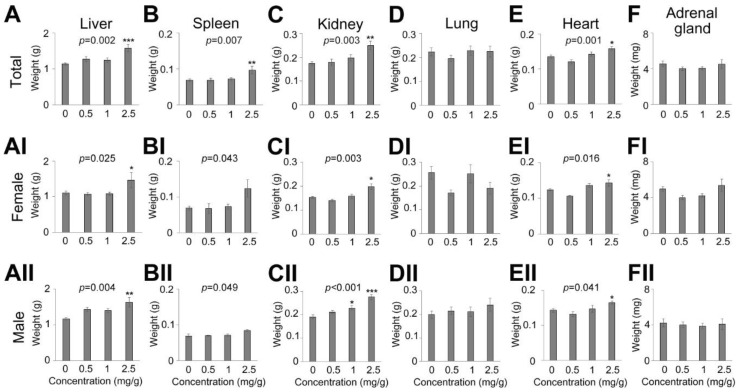
Effects on mouse organ weights by *Dendropanax trifidus* (DT) sap administration in mice. DT sap of the indicated concentrations (0, 0.5, 1, 2.5 mg/g for DT sap weight/body weight) was given to 9-week-old ICR mice (N = 4–5 per each group) by oral gavage every day for 14 days. Organ weights at day 14 depending on the concentration are shown: (**A**,**AI**,**AII**) liver; (**B**,**BI**,**BII**) spleen; (**C**,**CI**,**CII**) kidney; (**D**,**DI**,**DII**) lung; (**E**,**EI**,**EII**) heart; (**F**,**FI**,**FII**) adrenal gland. (**A**–**F**) Total; (**AI**–**FI**) female; (**AII**–**FII**) male. Indicated *p* values are derived from a group difference in one way ANOVA. The significance of post hoc analyses are indicated with asterisks. *, *p* < 0.05; **, *p* < 0.01; ***, *p* < 0.001. Bars indicate mean ± s.e.m.

To examine the effects of the concentration on tissue weight in a multiple administration regime, mouse tissues were collected and measured at day 14 (Figure 3). Using a one-way ANOVA against total organ weights, significant differences were found in the liver (*p* = 0.002, Figure 3A), spleen (*p* = 0.007, Figure 3B), kidney (*p* = 0.003, Figure 3C), heart (*p* = 0.001, Figure 3E), and with respect to concentration. By post hoc analysis (Holm–Sidak method), significant differences were found to be derived from one concentration (i.e., the highest concentration, 2.5 mg/g, *p* = 0.002 for kidney, 0.031 for heart, <0.001 for liver, 0.006 for spleen). There were no significant differences in the tissue weights at other concentrations. In addition, increasing DT sap concentration did not affect the lung and adrenal tissue weights. 

Analyzing by gender, the weights of female liver (one way ANOVA, *p* = 0.025, Figure 3AI), spleen (one way ANOVA, *p* = 0.043, Figure 3BI), kidney (one way ANOVA, *p* = 0.003, Figure 3CI), heart (Kruskal–Wallis one way ANOVA on Ranks, *p* = 0.016, Figure 3EI) were significantly changed and post hoc analysis revealed that these were due to the highest concentration of 2.5 mg/g (Holm–Sidak method, *p* = 0.039 for liver, *p* = 0.058 for spleen, *p* = 0.012 for kidney; Dunn’s Method, *p* = 0.035 for heart). There were no significant weight differences in the other concentrations. In female lung and adrenal glands, no significant weight differences were found in all the concentrations. 

In male mouse tissues, one-way ANOVA analysis showed significant differences in liver (*p* = 0.004, Figure 3AII), spleen (*p* = 0.049, Figure 3BII), kidney (*p* < 0.001, Figure 3CII), heart (*p* = 0.041, Figure 3EII) by DT sap concentrations, and subsequent post hoc analysis (Holm–Sidak method) showed that the difference was mainly derived from the 2.5 mg/g concentration, although differences were also found at 1 mg/g in kidney (liver, *p* = 0.003 (0 vs. 2.5 mg/g); kidney, *p* < 0.001(0 vs. 2.5 mg/g), *p* = 0.021 (0 vs. 1 mg/g); heart, *p* = 0.034 (0.5 vs. 2.5 mg/g)). There were no significant differences in these tissue weights in other concentrations. In male lung and adrenal glands, no differences were found in all the concentrations. 

### 3.3. Reduction in Blood Glucose by Dendropanax trifidus Sap Injection

**Figure 4:**
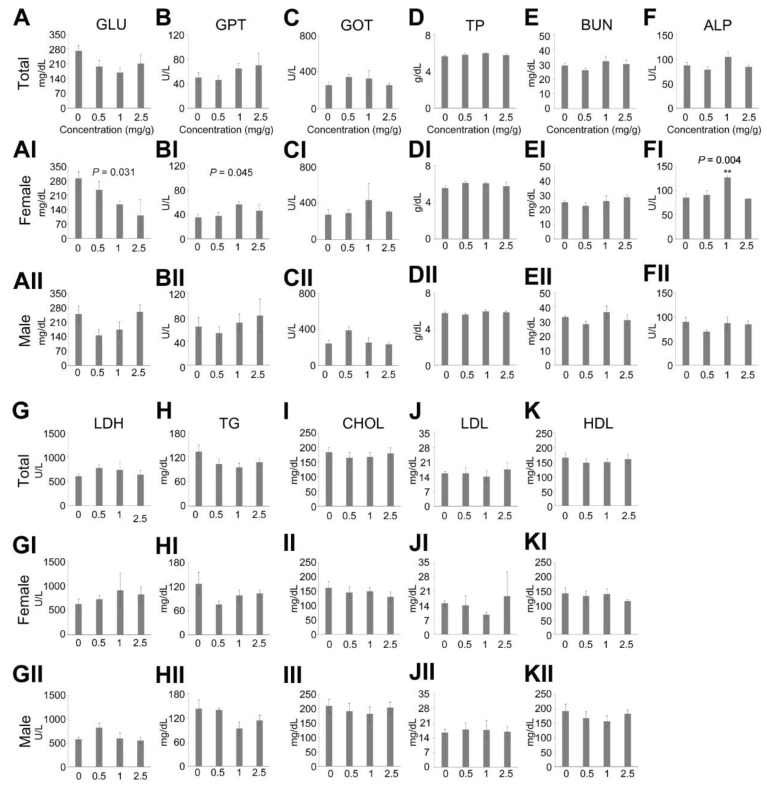
Effects on blood chemistry by *Dendropanax trifidus* (DT) sap administrations in mice. DT sap of the indicated concentrations was given to 9-week-old ICR mice (N = 4–5 per each group) by oral gavage every day for 14 days. Blood chemistry of following items was investigated: (**A**) GLU; (**B**) GPT; (**C**) GOT; (**D**) TP; (**E**) BUN; (**F**) ALP; (**G**) LDH; (**H**) TG; (**I**) CHOL; (**J**) LDL; (**K**) HDL. Data of total (**A**–**K**), female (**AI**–**KI**), male (**AII**–**KII**) mice are shown. Indicated *p* values are derived from a group difference in one way ANOVA. The significance of post hoc analysis is indicated with asterisks: **, *p* < 0.01. Bars indicate mean ± s.e.m.

**Figure 5:**
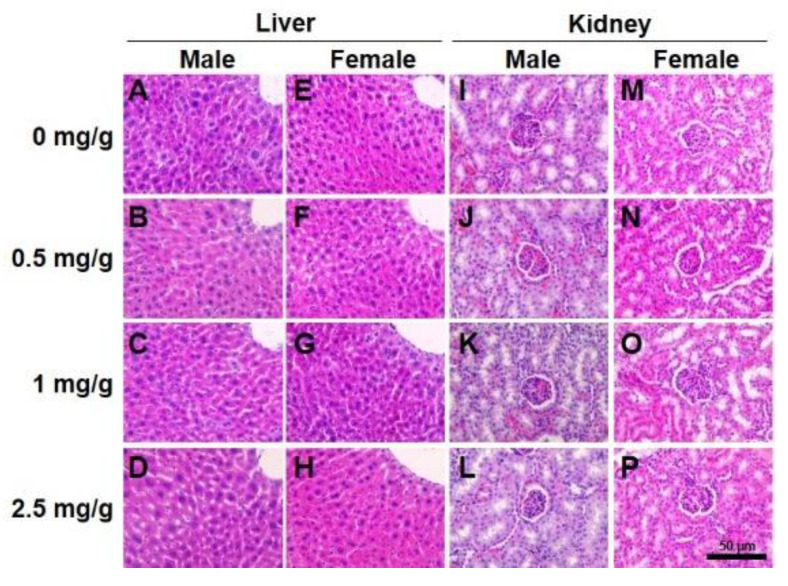
Effects on histopathological morphology of liver and kidney by *Dendropanax trifidus* (DT) sap administration in mice. DT sap of the indicated concentrations (0, 0.5, 1, 2.5 mg/g for DT sap weight/body weight) was given to 9-week-old ICR mice (N = 5 per each group) by oral gavage every day for 14 days. H&E staining was performed for the following tissues: (**A**–**D**) male liver; (**E**–**H**) female liver; (**I**–**L**) male kidney; (**M**–**P**) female kidney. Representative pictures are shown. All pictures are provided in Figure S2 and Figure 3. Scale bar, 50 μm.

Next, we investigated the effect of DT sap concentration on blood chemistry after 14 days of daily oral administration (Figure 4). In total mouse blood chemistry analysis, there was a tendency of reduction in blood GLU with respect to increasing DT sap concentration (One way ANOVA, *p* = 0.060, Figure 4A). Other components in the blood chemistry did not show this tendency. In addition, when the data was individually analyzed by *t*-test in comparison with vehicle (0 mg/g)-injected mice, mice treated with 0.5 mg/g and 1 mg/g exhibited a tendency of reduction (*p* = 0.0628) and a significant reduction (*p* = 0.005), respectively. In female mice, there was a significant reduction in GLU corresponding with the increase of DT sap concentrations (One way ANOVA, *p* = 0.031, Figure 4AI). In addition, significant changes in GPT and ALP were observed (*p* = 0.045 and 0.004, respectively) and subsequent post hoc analysis showed a significant increase in ALP at 1 mg/g (*p* = 0.006, Figure 4FI), but not in GPT (Figure 4BI). There were no significant changes in the other parameters (GOT, TP, BUN, LDH, TG, CHOL, LDL and HDL) with concentration (Figure 4CI–KI). In addition, LDL showed a significant reduction in 1 mg/g compared to vehicle control in *t*-test (*p* = 0.01, Figure 4JI). Unlike female mice, no significant correlation was found in GLU in males (Kruskal–Wallis one way ANOVA on Ranks, *p* = 0.093), although GLU levels at 0.5 mg/g DT sap were reduced about half compared to the vehicle control (Figure 4AII). No significant changes were found in the other parameters in males (Figure 4BII~KII). In hematoxylin and eosin staining of mouse liver and kidney after 14 days of injection, no specific histopathological changes were observed by DT sap administration in both male and female mice (Figures 5, S2 and S3, N = 5 per each concentration). To summarize, (1) DT sap did not affect mouse survival, body weight, tissue weight, blood chemistry and histochemistry at the concentration of ≤0.5 mg/g (Table S2), therefore the concentration of ≤0.5 mg/g is recommended for long term stable administration in mice. (2) A significant reduction was found in blood GLU with respect to increasing DT sap concentration in female mice (Figure 4AI), suggesting the potential effects of DT sap on GLU metabolism. 

### 3.4. Effects of Dendropanax trifidus Sap on AMPK-Mediated Signaling

**Figure 6:**
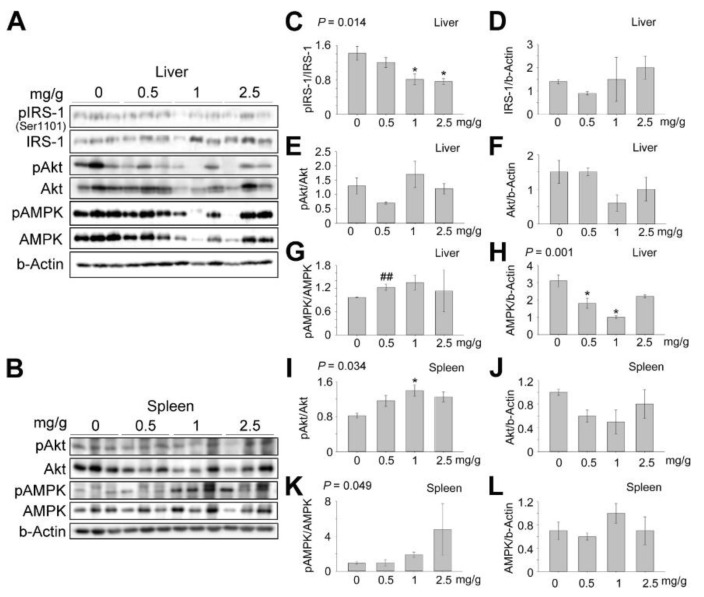
Effects on AMPK-mediated signaling after two weeks of daily oral administration of *Dendropanax trifidus* (DT) sap. DT sap of the indicated concentrations (0, 0.5, 1, 2.5 mg/g for DT sap weight/body weight) was given to 9-week-old ICR mice by oral gavage every day for 14 days. Western blot analysis on livers (**A**,**C**–**H**) or spleens (**B**,**I**–**L**) of the treated female mice. Blots of liver (**A**) or spleen (**B**) samples were incubated with antibodies to pIRS-1(ser1101), IRS-1, pAkt, Akt, pAMPK, AMPK, β-Actin, as indicated. (C) Relative value of pIRS-1 intensity normalized by general IRS-1 (*p* = 0.014, one way ANOVA; * *p* = 0.038 (1 mg/g), * *p* = 0.030 (2.5 mg/g), compared with 0 mg/g, post hoc Holm–Sidak method). (**D**) Relative value of IRS-1 intensity normalized by β-Actin. *p* = 0.548, one way ANOVA. (**E**) Relative value of pAkt intensity normalized by general Akt. *p* = 0.157, one way ANOVA. (**F**) Relative value of Akt intensity normalized by β-Actin. *p* = 0.143, one way ANOVA. (**G**) Relative value of pAMPK intensity normalized by general AMPK. ^##^ *p* = 0.007, compared with 0 mg/g, Student’s *t*-test. (H) Relative value of AMPK intensity normalized by β-Actin. *p* = 0.001, one way ANOVA; * *p* = 0.016 (0.5 mg/g), * *p* = 0.001 (1 mg/g), compared with 0 mg/g, post hoc Holm–Sidak method. (**I**) Relative value of pAkt intensity normalized by general Akt. *p* = 0.034, one way ANOVA; * *p* = 0.039 (1 mg/g), compared with 0 mg/g, post hoc Holm–Sidak method. (**J**) Relative value of Akt intensity normalized by β-Actin. *p* = 0.262, one way ANOVA. (**K**) Relative value of pAMPK intensity normalized by general AMPK. *p* = 0.049, one way ANOVA. (**L**) Relative value of AMPK intensity normalized by β-Actin. *p* = 0.476, one way ANOVA. N = 3 (female) mice per group. 20 ug/lane. Bars indicate mean ± s.e.m. The significance of one-way ANOVA post-hoc tests is indicated with asterisks: *, *p* < 0.05.

To further investigate the effects of DT sap on GLU metabolism in female mice, Western blots were performed in liver tissues for several key signaling factors known to be involved in the regulation of blood GLU (Figures 6,  S4 and  S5). Previous studies showed that the phosphorylation of IRS-1 at Ser 1101 results in an inhibition of insulin signaling in the cell [24]. We observed that pIRS-1(Ser1101)/IRS-1 levels were significantly reduced with respect to increasing DT sap concentration in liver tissues (Figure 6A,C, *p* = 0.014, one way ANOVA). We noted that there was a non-significant slight increase in IRS/β-Actin levels with respect to increasing DT sap concentration (Figure 6D). As Akt activation is known to act downstream of insulin signaling, we also measured Akt and pAkt levels. The levels of pAkt/Akt appear to alter at 0.5, 1 mg/g dose, without significant differences (Figure 6E). Akt/β-Actin was not changed at 0.5 mg/g and decreased at 1 or 2.5 mg/g, without significant changes (Figure 6F). 

Hepatic AMPK activation is known to induce a suppression in gluconeogenesis [25]. To see if the AMPK pathway is activated by DT sap, AMPK phosphorylation was investigated. Compared to the vehicle control, 0.5 mg/g DT sap injection over 14 days significantly increased pAMPK/AMPK in the liver tissues (Figure 6A,G, Student’s *t*-test, *p* = 0.032). Higher concentrations also increased pAMPK/AMPK level, but without significant changes. In addition, the AMPK/β-Actin level was significantly altered in liver tissue on application of DT sap (Figure 6H, *p* = 0.001, one way ANOVA). Post hoc analysis showed significant reductions in AMPK/β-Actin at 0.5 and 1 mg/g (* *p* = 0.016 in 0 vs. 0.5 mg/g; * *p* = 0.001 in 0 vs. 1 mg/g, Holm–Sidak method). 

The spleen is related with GLU homeostasis, and its absence induces diabetes in the long term [26,27]. Therefore, GLU signaling factors were investigated in the spleen as well. IRS-1 signaling could not be measured reliably in the spleen sample. However, pAkt/Akt levels were significantly increased along with an increase in DT sap concentrations (Figure 6B,I, *p* = 0.034, one way ANOVA; * *p* = 0.039 in 0 vs. 1 mg/g, post hoc Holm–Sidak method). Akt/β-Actin was slightly reduced by DT sap administration without significant changes (Figure 6J). The level of pAMPK/AMPK was significantly increased with increasing DT sap concentration (Figure 6B,K, *p* = 0.049, one way ANOVA). The AMPK/β-Actin was not altered or slightly increased by the DT sap application in spleen tissues without significant differences (Figure 6L). GLU transporters such as Glut4 and Glut1 also contribute to regulate GLU metabolism by facilitating the transport of GLU [28]. However, there was no significant changes in Glut4 and Glut1 levels by DT sap administration in both tissues (Figure S4). 

### 3.5. Component Analysis of Dendropanax trifidus Sap

**Figure 7:**
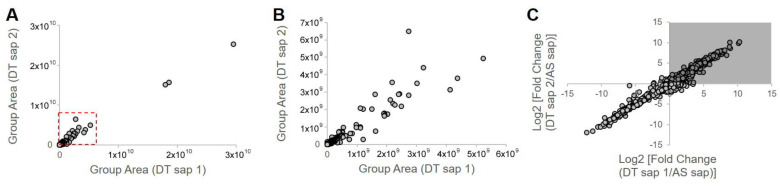
*Dendropanax trifidus* (DT) sap–derived chemicals identified by LC–MS/MS. Dots indicate 1080 chemicals identified by LC-MS/MS. Among them, 1041 chemicals were matched in more than one of seven annotation sources. Two batches of DT sap (indicated as DT sap 1, DT sap 2) and *Acer saccharum* (AS) sap were used. (**A**,**B**) Group area, which indicates area under curve of the intensity peak, of DT sap 1 and 2 are plotted. The plot region within the red dotted box is enlarged in (**B**). (**C**) The intensity of each chemical was compared between DT sap and AS sap to provide a fold change of DT sap/AS sap. X and Y axis indicate Log2 [fold change (DT sap 2/AS sap)] and Log2 [fold change (DT sap 1/AS sap)]. Chemical intensity is higher in DT sap than in AS sap in the shaded field (354 chemicals) and vice versa in the bright field (726 chemicals) in the graph. Representative chemicals in the shaded field, with high group area, are listed in Table 1.

To characterize the chemical composition of DT sap, two batches of DT sap extracts were subjected to LC-MS/MS. A total of 1080 chemicals were identified (Figure 7A,B). Among them 1041 chemicals were fully or partially matched in more than one of seven tested annotation sources. The top three chemicals with the highest peak area (outside of the dotted red box in Figure 7A) were (1E,5E,9E)-1,5,9-Trime-thyl-1,5,9-cyclododecatriene, caryophyllene oxide and curcumene (Table 1). Seven more chemicals with the next highest peak area (dots with high values in Figure 7B) were 3,5-di-tert-butyl-4-hydroxybenzaldehyde, capsidiol, estradiol (or 19-norandroestenedione), bis [2-(2-butoxyethoxy)ethyl]adipate, trenbolone, o-tert-octylphenol and 5-ethyl-3,8-dimethyl-1,7-dihydroazulene (Table 1). Next, to identify DT sap-specific components, LC-MS/MS was performed and compared with the sap from another tree (i.e., *Acer saccharum* (AS)). DT sap-specific components were screened by fold change analysis with AS sap, and the consistency of the component analysis was confirmed by repeat experiments using different batches (Figure 7C). In the graph of Figure 6C, dots of the shaded field (upper right quadrant) represent chemicals which are specifically abundant in DT sap compared to the AS sap, while dots of the other regions are chemicals which are specifically abundant in AS sap compared to DT sap, or unchanged. The intensity of 354 chemicals were higher in DT sap than in AS sap, while that of 726 chemicals were lower in DT sap. The top ten chemicals with the highest fold change was farnesol, gamfexine, (8z,11z,14z)-heptadecatrienoic acid, (1E,5E,9E)-1,5,9-Trime-thyl-1,5,9-cyclododecatriene, o-tert-octylphenol, cumene, α-Eleostearic acid, oleanolic aldehyde, 2,6-Dimethyl-4-nonylphenol and 2,6-Di-tert-butyl-4-(dimethylaminome-thyl)phenol (Table 1). In addition to the above-mentioned chemicals, representative chemicals abundant in DT sap include 2-hydroxyestradiol, dienogest, isotretinoin, linoleic acid and costunolide (Table 1).

## 4. Discussion

The results of our toxicity study on survival ratio, body weight, organ weight, blood chemistry and histochemistry show that DT sap concentrations below 0.5 mg/mL were safe for long-term administration (Table S2). We also found that blood GLU levels were gradually reduced with increased dosage of DT sap in female mice, and that the AMPK-mediated signaling was altered by DT sap administration in female mouse liver and spleen tissues. In addition, bioactive chemicals were identified from DT sap by LC-MS/MS.

While a significant dose-dependent reduction in blood glucose was observed in female mice, it was not observed in male mice (Figure 4AI,AII). However, 0.5 mg/g DT sap administration also reduced the mean value of blood glucose in males compared to the control, although it was not statistically significant, implying a possible gender difference in dose-response. Therefore, we cannot exclude the possibility of hypoglycemic effects of DT sap on male mice as well, warranting further studies. Reduction in blood GLU with corresponding increases of DT sap concentration (Figure 4AI) may be related with the activation of the insulin signaling pathway [29] or AMPK signaling pathway [30]. Here, phosphorylation of IRS-1 and Akt for the insulin signaling pathway and phosphorylation of AMPK for the AMPK signaling pathway were investigated. Phosphorylation of IRS-1 (Ser1101) inhibits insulin signaling [24], therefore the reduction of pIRS-1(Ser1101)/IRS-1 by DT sap should result in a corresponding decrease in blood GLU, which is observed here (Figure 6C). The level of pAkt/Akt, a downstream signaling factor of IRS-1, appears to increase according to DT sap concentration (Figure 6I). These results suggest that DT sap appears to affect blood GLU regulation via activation of the insulin signaling pathway. Previous findings showed that the activation of AMPK suppresses gluconeogenesis [25]. In our study, pAMPK/AMPK levels were significantly increased at low DT sap concentrations (0.5 mg/g) in liver tissues (Figure 6G), and exhibited a significant increase in conjunction with increasing DT sap concentration in spleen tissues (Figure 6K). Therefore, reduced GLU production may also contribute to blood GLU reduction (Figure 4AI).

We observed that pAMPK/AMPK, a key metabolic regulator, exhibited a common tendency of increase with respect to increasing DT sap concentrations in both liver and spleen tissues (Figure 6G,K), In contrast, pAkt/Akt levels responded differently in liver and spleen tissues. While pAkt/Akt levels did not show a consistent change in liver tissue (Figure 6E), the pAkt/Akt levels in the spleen tissue showed an increase, with respect to increasing DT sap concentration (Figure 6I). At this stage, we cannot explain the divergence in pAkt/Akt between liver and spleen tissues, but this will be a matter of future investigation.

The β-actin-normalized AMPK level was significantly altered by DT sap and post hoc analysis showed that it was significantly reduced in 0.5 and 1 mg/g DT sap (Figure 6H). The other total protein levels of IRS, Akt in liver as well as Akt, AMPK in spleen exhibited slight reductions at some dosage, but without significant differences (Figure 6D,F,J,L). The reduction of total AMPK protein expression (Figure 6H) might be a form of negative feedback to preserve cellular homeostasis by preventing the overactivation of AMPK signaling (Figure 6G,K) induced by long-term DT sap administration. High dosage (2.5 mg/g) might disrupt cellular homeostasis, resulting in significant organ weight changes (Figure 3) and lower survival rate (Figure 2), observed here.

Interestingly, chemicals enriched in DT sap as analyzed by LC-MS/MS included estradiol, its related metabolites or structurally similar substances (e.g., 2-hydroxyestradiol, trenbolone, dienogest) (Table 1). It was previously shown that estradiol injections reduced fasting blood GLU levels in non-obese C57BL/6N mice with short-term ovariectomy, while AMPK was activated and gluconeogenic gene expression was reduced in liver tissues [31]. A 4-week subcutaneous injection of estradiol to ovariectomized female mice with STZ-induced type 1 diabetes significantly suppressed blood GLU level and increased plasma insulin, suggesting protective effects of estradiol against STZ-induced diabetes [32]. Besides estradiol, various components were identified from DT sap, such as caryophyllene oxide, curcumene, 3, 5-di-tert-butyl-4-hydroxybenzaldehyde, trenbolone, farnesol, dienogest, 2-Hydroxyestradiol, isotretinoin, linoleic acid and costunolide (Table 1). Among them, 2-hydroxyestradiol is a metabolite of estradiol and directly activates AMPK in C2C12 myotubes [33], which attenuates the development of obesity and decreases the severity of diabetes [34]. A black pepper extract with a high content of caryophyllene improved GLU uptake in C2C12 myotubes [35]. Farnesol induced a significant reduction of postprandial hyperglycemia on alloxan-induced type 2 diabetic mice [36]. The function of linoleic acid relates with the regulation of body weight, and serum leptin in subjects with type 2 diabetes [37]. These components have potentials to affect GLU metabolism, which may be involved in the reduction of blood GLU by DT sap observed here. However, to identify the actual bioactive components, in vitro screening and in vivo tests using isolated compounds are required, which is expected as a follow-up study.

## 5. Conclusions

In this study, we examined the in vivo safety, hypoglycemic function and component analysis of DT sap. Toxicity tests in vivo confirmed safety in mice at less than 0.5 mg/g when administered for longer than 14 days. Our results showed that DT sap exhibited the effect of reducing blood GLU and that DT sap can be linked to mechanisms involved in blood GLU regulation at least partially by modulating IRS, Akt and AMPK signaling in liver and spleen tissues. From the LC-MS/MS analysis, DT sap included various bioactive chemicals, suggesting their potential contribution to the function of DT sap, warranting further studies. In future, studies on the effects of DT sap on diabetes and the identification of effective components are expected.

## Figures and Tables

**Table 1:** Representative chemical components of *Dendropanax trifidus* sap ethanol extracts identified by Liquid Chromatography-tandem Mass Spectrometry.

Molecular Formula	Suggested Compound	Synonyms (Pubchem)	Suggested Structure (Pubchem)	Annotation		MS/MS (Database Search Score)	Molecular Weight	Retention Time (min)	Max. Area (Arbitrary Unit, 1.0 × 10^8^)	Mean Group Area (Arbitrary Unit, 1.0 × 10^8^)	Mean [Log2 Fold Change (DT Sap/AS Sap)]
				Full	Partial						
C15H24	(1E,5E,9E)-1,5,9-Trimethyl-1,5,9-cyclododecatriene	1,5,9-Trimethyl cyclododecatriene	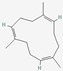	2	3	94.8 *	204.19	21.962	260.7	274.2	8.79
C15H24O	Caryophyllene oxide	(-)-Caryophyllene oxide; beta-Caryophyllene oxide	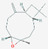	3	3	93.6	220.18	22.201	136.9	171.4	6.51
C15H22	Curcumene	Alpha-Curcumene; 2-Methyl-6-p-tolyl-2-heptene	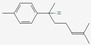	2	2	96.2 *	202.17	21.832	130.7	165.2	5.69
C15H22O2	3,5-di-tert-butyl-4-hydroxybenzaldehyde	3,5-Di-t-butyl-4-hydroxybenzaldehyde	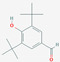	2	3	85 *	234.16	20.281	63.91	50.75	3.59
C15H24O2	Capsidiol	(1R,3R,4S,4aR,6R)-6-Isopropenyl-4,4a-dimethyl-1,2,3,4,4a,5,6,7-octahydro-1,3-naphthalenediol	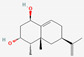	2	2	87.4 *	236.18	21.501	51.11	40.85	6.98
C18H24O2	19-Norandrostenedione	Estr-4-ene-3,17-dione; 19-Norandrost-4-ene-3,17-dione; Norandrostenedione	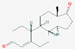	4	2	87.2	272.18	22.014	36.15	38.09	6.44
	Estradiol	beta-Estradiol; 17beta-Estradiol	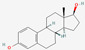								
C22H42O8	Bis [2-(2-butoxyethoxy)ethyl] adipate	Dibutoxyethoxyethyl adipate	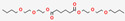	2	1	68.8 *	434.29	21.792	36.42	36.30	1.49
C18H22O2	Trenbolone	17beta-Trenbolone; Trienbolone; 17-beta-Hydroxyestra-4,9,11-trien-3-one	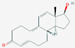	3	3	84.1	270.16	21.313	39.92	28.62	6.43
C14H22O	o-tert-Octylphenol	t-octylphenol; 2-(2,4,4-trimethylpentan-2-yl)phenol	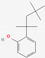	2	1	80.6 *	206.17	21.866	31.28	27.76	7.79
C14H18	5-Ethyl-3,8-dimethyl-1,7-dihydroazulene	5-Ethyl-3,8-dimethyl-1,7-dihydroazulene	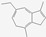	2	0	83.8 *	186.14	21.163	29.03	26.66	6.87
C15H26O	Farnesol	trans,trans-Farnesol; (2E,6E)-Farnesol; (E,E)-Farnesol	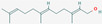	2	2	88.3 *	222.20	22.165	25.87	22.67	10.23
C17H27N	Gamfexine	3-cyclohexyl-N,N-dimethyl-3-phenylpropan-1-amine	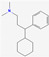	2	0	64.1 *	245.21	22.155	19.50	23.41	9.99
C17H28O2	(8Z,11Z,14Z)-heptadecatrienoic acid	Norlinolenic acid	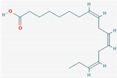	2	1	86.3 *	264.21	20.475	12.91	10.55	8.88
C9 H12	Cumene	Isopropylbenzene; 2-Phenylpropane	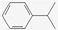	2	2	94.8 *	120.09	21.986	21.04	18.09	7.66
C18H30O2	α-Eleostearic acid	alpha-eleostearic acid; Margarolic acid	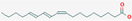	2	3	92.8	278.22	22.430	1.716	1.519	7.46
C30 H48 O2	oleanolic aldehyde	3beta-hydroxyolean-12-en-28-al	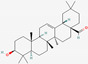	3	1	88.6 *	440.36	23.402	0.7476	0.5755	7.36
C17H28O	2,6-Dimethyl-4-nonylphenol	Phenol, 2,6-dimethyl-4-nonyl-	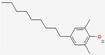	2	1	91.6 *	248.21	21.662	2.387	2.331	7.27
C17H29NO	2,6-Di-tert-butyl-4-(dimethylaminomethyl)phenol	N,N-dimethyl-3,5-di-tert-butyl-4-hydroxybenzylamine	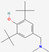	2	0	78.7 *	263.22	21.865	4.091	3.238	7.20
C18H24O3	2-Hydroxyestradiol	2-OH-Estradiol; 2-hydroxy-estradiol; Estra-1,3,5(10)-triene-2,3,17beta-triol	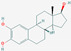	2	2	81.6 *	288.17	21.268	22.30	17.78	4.08
C20H25NO2	Dienogest	Dienogestrel; Dinagest; Endometrion	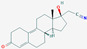	3	0	63.1	311.19	21.276	22.67	16.19	6.85
C20H28O2	Isotretinoin	13-cis-Retinoic acid; 3-cis-Vitamin A acid; Accutane	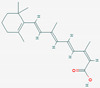	4	3	81.2	300.21	22.691	7.111	5.430	0.99
C18H32O2	Linoleic acid	Linolic acid; Telfairic acid; cis,cis-Linoleic acid	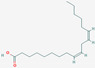	4	0	94.7 *	280.24	21.686	3.033	2.935	6.45
C15H20O2	Costunolide	(+)-costunolide; Costunlide; Costus lactone	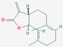	2	2	94.8 *	232.16	21.149	5.355	2.636	4.68

Representative chemicals are listed. Annotation sources: Predicted Compositions; mzCloud Search; mzVault Search; Metabolika Search; ChemSpider Search; BioCyc Search; MassList Search. MS/MS (database search score): mzCloud Best Match; *, mzCloud Best Sim. Match. Abbreviations: DT, *Dendropanax trifidus*; AS, *Acer saccharum*.

## Data Availability

The data presented in this study are available on request from the corresponding author.

## Supplementary Materials

The following are available online at https://www.mdpi.com/article/10.3390/nu13124332/s1. Figure S1: Survival rates, body and organ weights by a single oral administration of *Dendropanax trifidus* (DT) sap, Figure S2: Morphology of liver tissue by *Dendropanax trifidus* (DT) sap administrations to mice, Figure S3: Morphology of kidney tissue by *Dendropanax trifidus* (DT) sap administrations to mice, Figure S4: Effects of *Dendropanax trifidus* (DT) sap administrations on the protein expression level of glucose transporters, Figure S5: Western blot Images of Figure 6, Table S1: Mouse number used in each experiment, Table S2: Summary of the concentration-dependent effects in vivo.
