# Supplementary material for: In Vivo Hypoglycemic Effects, Potential Mechanisms and LC-MS/MS Analysis of Dendropanax Trifidus Sap Extract
Source: Nutrients. 2021 Nov 30;13(12):4332. doi: 10.3390/nu13124332 (PMC8703777; doi:10.3390/nu13124332)
Supplement: Supplementary file 1 [file nutrients-13-04332-s001.zip › nutrients-1435958-supplementary.pdf]

Supplementary Materials

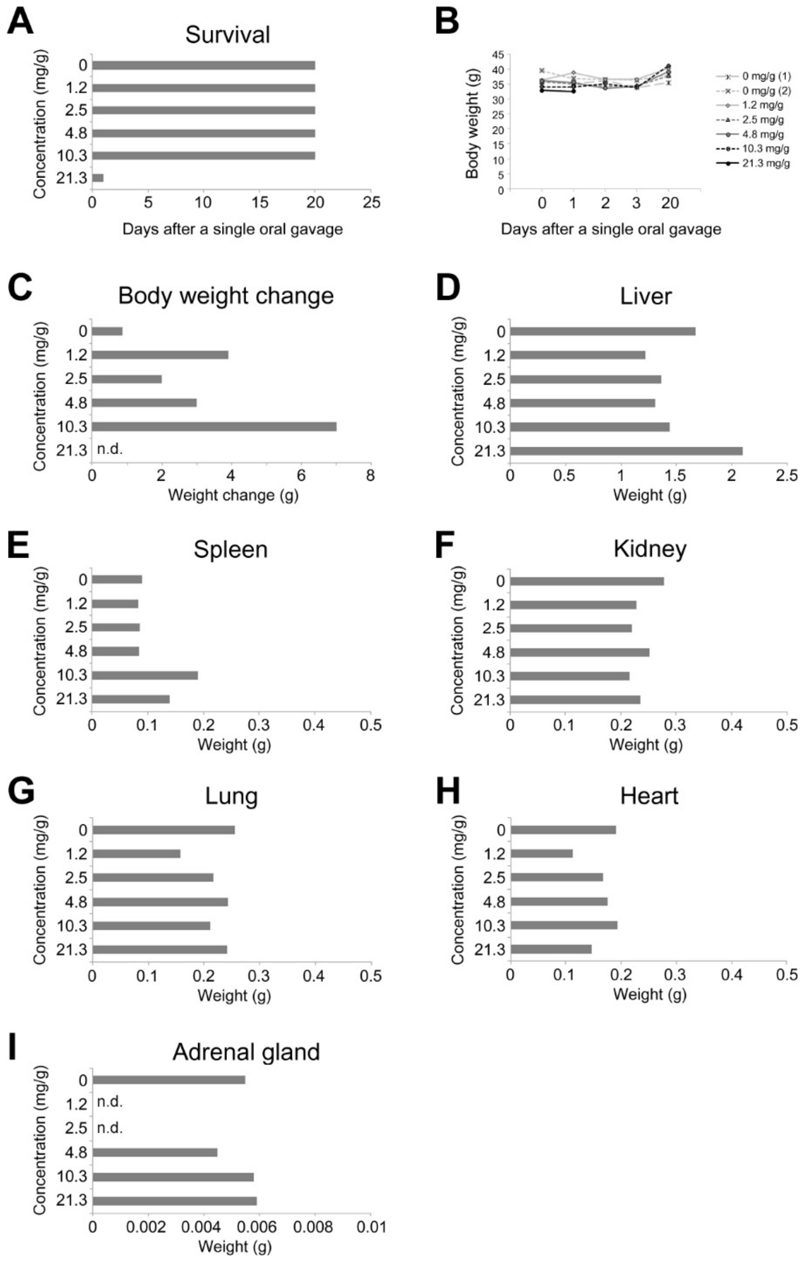

Figure S1. Survival rates, body and organ weights by a single oral administration of *Dendropanax trifidus* (DT) sap. (A) Survival days after a single oral administration. (B) Body weight after a single oral administration at the indicated days. (C-I) Changes of body and organ weights at day 20 (mg/g for DT sap weight/body weight): (C) body weight change; (D) liver weight; (E) spleen weight; (F) kidney weight; (G) lung weight; (H) heart weight; (I) adrenal gland weight. N=1. n.d., no data.

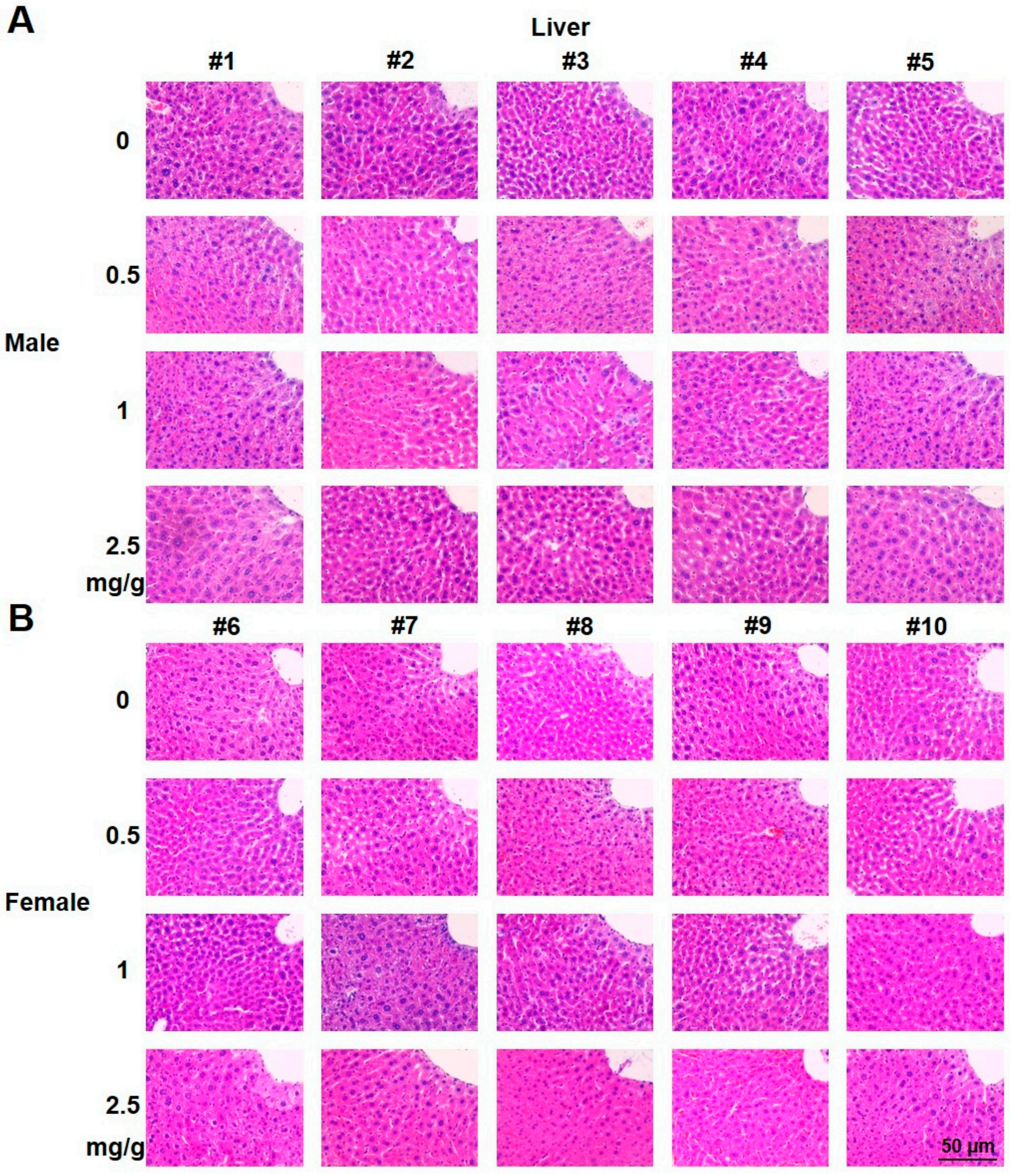

Figure S2. Morphology of liver tissue by *Dendropanax trifidus* (DT) sap administrations to mice. DT sap of the indicated concentrations (0, 0.5, 1, 2.5 mg/g for DT sap weight/body weight) were given to 9 weeks old-ICR mice (N = 5 per each concentration) by oral gavage every day for 14 days. H&E staining was performed for liver tissue. (A) male mice; (B) female mice. #1~#10 indicates each mouse number. Scale bar, 50 μm.

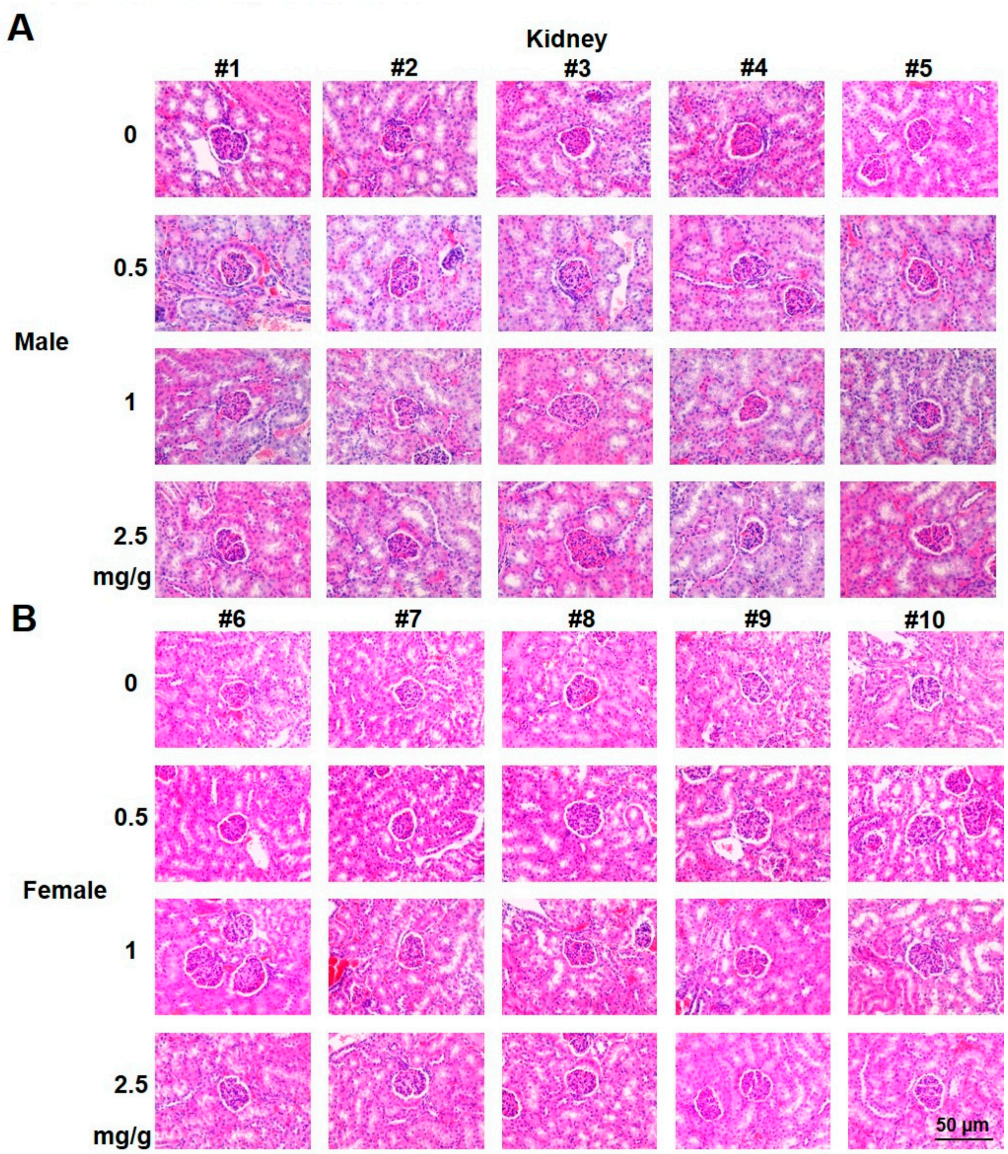

Figure S3. Morphology of kidney tissue by *Dendropanax trifidus* (DT) sap administrations to mice. DT sap of the indicated concentrations (0, 0.5, 1, 2.5 mg/g for DT sap weight/body weight) were given to 9 weeks old-ICR mice (N = 5 per each concentration) by oral gavage every day for 14 days. H&E staining was performed for kidney. (A) male mice; (B) female mice. #1~#10 indicates each mouse number. Scale bar, 50 μm.

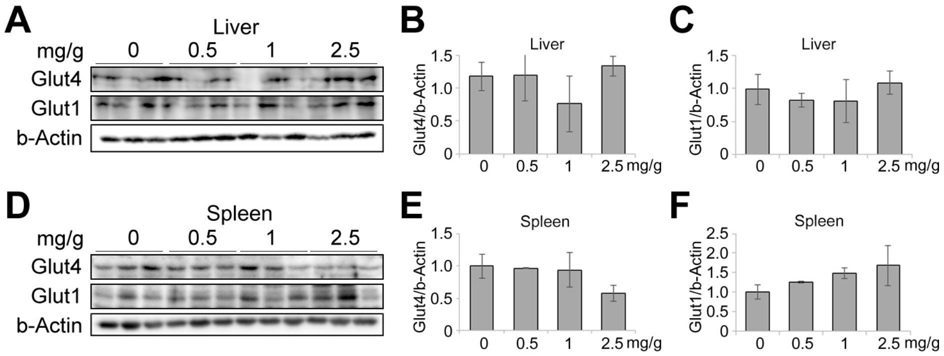

Figure S4. Effects of *Dendropanax trifidus* (DT) sap administrations on the protein expression level of glucose transporters. DT sap of the indicated concentrations (0, 0.5, 1, 2.5 mg/g for DT sap weight/body weight) were given to 9 weeks old-ICR mice by oral gavage every day for 14 days. Western blot analysis was performed on liver (A-C) and spleen (D-F) tissues against antibodies to Glut1, Glut4 and b-Actin, as indicated. (B, E) Relative value of Glut4 intensity normalized by b-Actin. (C, F) Relative value of Glut1 intensity normalized by β-Actin. N = 3 (female) mice per group. 20 ug/lane. Bars indicate mean ± s.e.m.

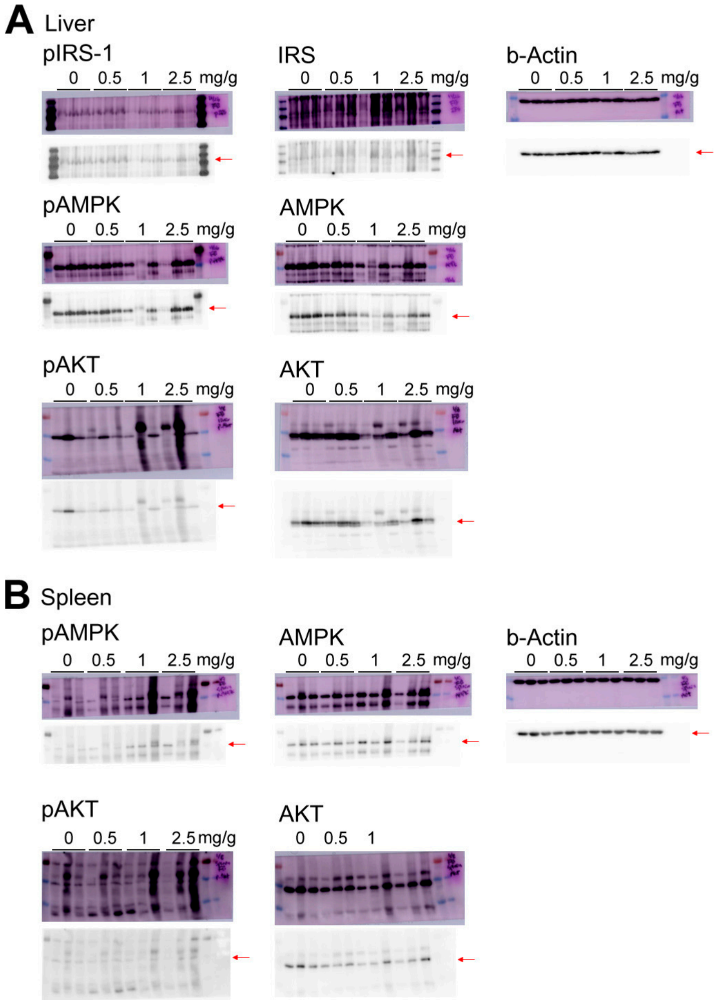

Figure S5. Western blot Images of Figure 6. (A) Western blot images of liver samples. (B) Western blot images of spleen samples. Red arrows indicate the targeted molecular weight.

Table S1. Mouse number used in each experiment.

Figure 2, 3.		
Conc. mg/g	Sex	Number of mouse
0	male	5
0.5		5
1		5
2.5		5
0	female	4
0.5		4
1		4
2.5		4

Figure 4.												
Conc.	Sex	Number of mouse										
mg/g		GOT	GPT	TP	BUN	ALP	LDH	HDL	LDL	TG	CHOL	GLU
0	male	5	5	5	5	5	5	4	4	4	4	4
0.5		5	5	5	5	5	5	3	3	3	3	3
1		5	5	5	5	5	5	5	5	4	5	5
2.5		4	4	4	4	4	4	4	4	3	4	4
0	female	4	4	4	4	4	4	4	4	4	4	4
0.5		4	4	4	4	4	4	4	4	4	4	4
1		4	4	4	4	4	4	4	4	4	4	4
2.5		3	3	3	3	3	3	3	3	3	3	3

Figure 5, Supplementary material 2, 3.			
Conc.	Sex	Number of mouse	
mg/g		Liver	Kidney
0	male	5	5
0.5		5	5
1		5	5
2.5		5	5
0	female	5	5
0.5		5	5
1		5	5
2.5		5	5

Figure 6, Supplementary material 4.		
Conc.	Sex	Number of mouse

mg/g		Liver	Spleen
0	female	3	3
0.5		3	3
1		3	3
2.5		3	3

**Supplementary material 1.**	
Conc. mg/g	Number of mouse
0	2
1.2	1
2.5	1
4.8	1
10.3	1
21.3	1

Table S2. Summary of the concentration-dependent effects in vivo. "O" shows a non-significant change, while "X" indicates a significantly different value from that of the vehicle control at the indicated concentration.

Sex		Female				Male			
Concentration (mg/g)		0	0.5	1	2.5	0	0.5	1	2.5
Survival rate		O	O	O	X	O	O	O	X
Body weight		O	O	O	O	O	O	O	O
Organ weight	Liver	O	O	O	X	O	O	O	X
	Spleen	O	O	O	O	O	O	O	O
	Kidney	O	O	O	X	O	O	X	X
	Lung	O	O	O	O	O	O	O	O
	Heart	O	O	O	X	O	O	O	X
	Adrenal gland	O	O	O	O	O	O	O	O
Blood chemistry	GLU	O	O	O	O	O	O	O	O
	GPT	O	O	O	O	O	O	O	O
	GOT	O	O	O	O	O	O	O	O
	TP	O	O	O	O	O	O	O	O
	BUN	O	O	O	O	O	O	O	O
	ALP	O	O	X	O	O	O	O	O
	LDH	O	O	O	O	O	O	O	O
	TG	O	O	O	O	O	O	O	O
	CHOL	O	O	O	O	O	O	O	O
	LDL	O	O	O	O	O	O	O	O
	HDL	O	O	O	O	O	O	O	O
Histochemistry	Liver	O	O	O	O	O	O	O	O
	Kidney	O	O	O	O	O	O	O	O
